# Therapie der Osteoporose bei systemischer Sklerose: Herausforderungen und Strategien im klinischen Alltag

**DOI:** 10.1007/s00393-025-01739-4

**Published:** 2025-10-10

**Authors:** Nils Schulz, Ulf Müller-Ladner, Philipp Klemm

**Affiliations:** Campus Kerckhoff, Abteilung für Rheumatologie, Immunologie, Osteologie und physikalische Medizin, Justus-Liebig-Universität Giessen, Benekestr. 2–8, 61231 Bad Nauheim, Deutschland

**Keywords:** Antiresorptiva, Fraktur, Knochendichte, Kollagenose, Trabecular Bone Score, Antiresorptive drugs, Bone mineral density, Connective tissue disease, Fracture, Trabecular bone score

## Abstract

**Hintergrund:**

Die systemische Sklerose (SSc) ist eine komplexe Autoimmunerkrankung mit multiorganischer Manifestation und erheblicher Morbidität und Mortalität. Aufgrund dessen sind SSc-Patienten übermäßig häufig von Osteoporose betroffen, die im Kontext der SSc bislang unzureichend charakterisiert ist und im Praxisalltag oftmals untergeht.

**Ziel:**

Darstellung der aktuellen Evidenzlage zur Epidemiologie, pathophysiologischen Mechanismen, diagnostischen Besonderheiten sowie therapeutischen Herausforderungen der Osteoporose im Kontext der SSc.

**Methodik:**

Narrative Übersicht relevanter Primär- und Sekundärliteratur (2003–2025) aus PubMed, Embase und aktuellen Leitlinienquellen mit Fokus auf osteologische Aspekte bei SSc.

**Ergebnisse:**

Patienten mit SSc zeigen eine signifikant erhöhte Prävalenz von Osteoporose und Frakturen. Die SSc aggraviert über Vaskulopathie, Fibrose wie auch Autoimmunität/Entzündungsaktivität Risikofaktoren der Osteoporose wie postmenopausaler Status, Malnutrition und Untergewicht. Diagnostisch erweist sich der Trabecular Bone Score (TBS) als ergänzendes Verfahren zur Frakturrisikobeurteilung. Die SSc erschwert v. a. über die gastrointestinale Manifestation zudem die osteospezifische Therapie, insbesondere orale Bisphosphonate scheinen weniger gut zu wirken. Erste Hinweise auf die Wirksamkeit parenteraler Antiresorptiva liegen vor.

**Diskussion:**

Die Osteoporose bei SSc erfordert ein krankheitsspezifisches Risikomanagement und ein individualisiertes diagnostisches sowie therapeutisches Vorgehen. Weitere prospektive Studien sind notwendig, um evidenzbasierte Therapieempfehlungen zu etablieren.

## Abstract (en)

**Background:**

Systemic sclerosis (SSc) is a complex autoimmune disease with multiorgan involvement and substantial morbidity and mortality. As a result, patients with SSc are disproportionately affected by osteoporosis, which remains insufficiently characterized in the context of SSc and is often underrecognized in clinical practice.

**Objective:**

To present the current state of evidence regarding the epidemiology, pathophysiological mechanisms, diagnostic features, and therapeutic challenges of osteoporosis in the context of SSc.

**Methods:**

Narrative review of relevant primary and secondary literature (2003–2025) from PubMed, Embase, and current guideline sources, focusing on osteological aspects in SSc.

**Results:**

Patients with SSc exhibit a significantly increased prevalence of osteoporosis and fractures. SSc aggravates osteoporosis risk factors such as postmenopausal status, malnutrition and low body weight through vasculopathy, fibrosis, as well as autoimmunity/inflammatory activity. The trabecular bone score (TBS) serves as a useful adjunct in fracture risk assessment. Furthermore, SSc complicates, especially due to gastrointestinal involvement, osteospecific therapy, with oral bisphosphonates appearing less effective. Preliminary evidence supports the effectiveness of parenteral antiresorptive agents.

**Conclusion:**

Osteoporosis in SSc requires disease-specific risk assessment and an individualized diagnostic and therapeutic approach. Further prospective studies are needed to establish evidence-based treatment recommendations.

Die systemische Sklerose (SSc) ist eine komplexe Autoimmunerkrankung, die primär durch Fibrose, Mikroangiopathie und Autoimmunität gekennzeichnet ist. Während kutane, vaskuläre und/oder viszerale Manifestationen im klinischen Alltag im Vordergrund stehen, sollten die Auswirkungen auf den Knochenmetabolismus im Kontext der Langzeitbehandlung nicht unterschätzt und unbeachtet bleiben. Aktuelle Erkenntnisse weisen auf eine ausgeprägte Osteovulnerabilität dieser Patientengruppe hin. Klassische Risikofaktoren der Osteoporose liegen bei SSc akzentuiert vor. Angesichts der prognostischen Relevanz osteoporotischer Frakturen ist eine systematische Bewertung knochenbezogener Veränderungen bei SSc essenziell. Dieses Manuskript beleuchtet die aktuellen Erkenntnisse zur Epidemiologie, Pathophysiologie, Diagnostik und Therapie osteologischer Manifestationen bei SSc und diskutiert Implikationen für die rheumatologische Praxis basierend auf einer narrativen Literaturübersicht (2003–2025) mit gezielter Recherche in PubMed und Embase. Berücksichtigt wurden vor allem systematische Übersichtsarbeiten sowie – soweit verfügbar – prospektive klinische Studien; die Evidenzlage stützt sich jedoch überwiegend auf retrospektive Analysen und Querschnittsstudien, während randomisierte kontrollierte Studien zur Diagnostik und Therapie der Osteoporose bei SSc bislang nicht vorliegen. Spezifische Leitlinien zu diesem Themenfeld existieren derzeit nicht.

## Epidemiologie

Patienten mit SSc weisen überdurchschnittlich häufig eine Knochendichteminderung auf. In Metaanalysen fanden sich Prävalenzen von bis zu 29,3 % für eine Osteoporose und 50 % für eine Osteopenie [1–3]. Das berechnete Risiko für Osteoporose bei SSc ist etwa dreifach erhöht im Vergleich zu Kontrollgruppen (Odds-Ratio [OR] = 3,1, 95 % Konfidenzintervall [KI] 2,3–4,0) [1]. Besonders beachtenswert ist die signifikant niedrigere Knochenmineraldichte (KMD) in der Kohorte der prämenopausalen Frauen wie auch der Männer im Vergleich zur Allgemeinbevölkerung (∆KMD Gesamthüfte prämenopausale Frauen und Männer jeweils 53 mg/cm^2^) [3]. Die Prävalenz der Osteoporose bei Patienten mit SSc ist vergleichbar mit der bei Patienten mit rheumatoider Arthritis (RA) – einer Erkrankung, die ein fester Bestandteil des Risikoassessments nationaler und internationaler Leitlinien zur Osteoporose darstellt [4–6].

Die beobachtete Knochendichteminderung und das insgesamt erhöhte Frakturrisiko sind klinisch relevant. In einer Metaanalyse zur Knochengesundheit bei Patienten mit SSc zeigte sich eine relevante Erhöhung der Rate von osteoporotischen Frakturen. Insbesondere ist das Risiko für Wirbelkörperfrakturen (OR 10,4, 95 % KI [1,2–90,6]; *p* = 0,03) bei Patienten mit SSc erhöht [7]. Dabei erlitten die Patienten mit SSc osteoporotische Frakturen im Mittel in jüngerem Alter (67 vs. 75 Jahre; *p* = 0,005) als normalerweise in reinen postmenopausalen Kollektiven [8]. Weiterhin besonders hervorzuheben ist, dass die Frakturen mit einer signifikant erhöhten 1‑Jahres-Mortalität nach dem Frakturereignis assoziiert sind (13 % vs. 3 %; *p* = 0,006) [8].

## Pathophysiologie

### Allgemeine Aspekte der Osteoporose

Osteoporose ist eine systemische Skeletterkrankung, die durch reduzierte KMD und gestörte Mikroarchitektur zu erhöhter Frakturgefahr führt. Pathophysiologisch liegt ein Ungleichgewicht zwischen knochenauf- und -abbauenden Prozessen zugrunde [9]. Wichtige pathophysiologische Faktoren sind Östrogenmangel (insbesondere postmenopausal), chronische Entzündung und Erhöhung proinflammatorischer Zytokinspiegel, Alterung, Malnutrition, Untergewicht und Immobilisation [9], welche oftmals und teils additiv/gebündelt bei SSc-Patienten zu finden sind und durch die SSc als Grunderkrankung in ihrer Ausprägung bzw. dem Schweregrad verstärkt werden.

### Osteologische Risikofaktoren im Kontext der systemischen Sklerose

Menschen mit SSc entwickeln häufiger eine Osteoporose, wobei das genetische Risiko nicht gegenüber der Allgemeinbevölkerung erhöht ist [10]. Die klassischen Risikofaktoren der Osteoporose scheinen bei Patienten mit SSc aggraviert zum Tragen zu kommen. Dies ist bedingt durch die Pathogenese der SSc mit den drei Hauptfaktoren Vaskulopathie, Fibrose und gesteigerte Autoimmunität.

So zeigen Patientinnen mit SSc ein signifikant erhöhtes Risiko für eine vorzeitige Ovarialinsuffizienz und ein früheres Menopausenalter im Vergleich zur Allgemeinbevölkerung [11]. Zudem weisen SSc-Patienten höhere Spiegel proinflammatorischer Zytokine als Resultat der chronisch-entzündlichen Autoimmunerkrankung auf [12]. Malnutrition und ein erniedrigter Body-Mass-Index (BMI) sind bei SSc häufig und resultieren überwiegend aus der gastrointestinalen (GI) Beteiligung mit Mikrostomie, Dysphagie, Motilitätsstörungen und Malabsorption [13, 14]. Zusätzlich tritt regelhaft eine Dysbiose auf. Auch wenn dies für SSc bislang nicht untersucht wurde, zeigen andere Erkrankungen, dass eine gestörte Mikrobiota zu einer Schwächung der intestinalen Barrierefunktion, zur Förderung systemischer Inflammation und zu Veränderungen des Metabolismus beitragen kann – mit potenziell negativer Wirkung auf die Knochenhomöostase. Die bei SSc hochprävalente gastroösophageale Refluxkrankheit erfordert zudem häufig eine Langzeittherapie mit Protonenpumpeninhibitoren (PPI), welche die intestinale Kalziumresorption vermindern und in Metaanalysen mit reduzierter KMD, gestörter Mikroarchitektur und erhöhter Frakturrate assoziiert sind [14–16].

Zusätzlich kommen krankheitsassoziierte Faktoren wie Vaskulopathie, Autoimmunität und organspezifische Manifestationen neben der GI-Beteiligung hinzu. Nicht nur die Organmanifestation als solche, sondern auch deren medikamentöse Therapie kann einen negativen Einfluss auf die Knochengesundheit nehmen (wie z. B. PPI). Auch wenn bei SSc deutlich seltener indiziert, ist die negative Wirkung von Glukokortikoiden auf die Knochenmikroarchitektur auch bei geringeren Dosen als bei anderen entzündlichen Erkrankungen wie beispielsweise der RA belegt [17].

Eine Übersicht der klassischen und krankheitsassoziierten Risikofaktoren bei der SSc bietet Tab. 1.

**Tab. 1:** Klassische, SSc-assoziierte und medikamentöse Risikofaktoren für Osteoporose

Kategorie	Risikofaktor	Anmerkung
Klassisch	Alter	Unveränderlich
	(Postmenopausaler) Östrogenmangel	Frühere Menopause und vermehrte Ovarialinsuffizienz bei SSc
	Malnutrition/Untergewicht	Sekundär durch gastrointestinale Manifestation, häufig unterschätzt
	Immobilisation	Schmerzen und Fatigue durch muskuloskeletale Manifestation, Hypoxie bei ILD
	Vitamin-D-Mangel	Prävalenz bis zu 97 %, verstärkt durch Malabsorption und Dermatosklerose
SSc-assoziiert	Mikroangiopathie, DU	Reduzierte Knochenperfusion, trabekuläre Schäden
	Autoantikörper	Lediglich Assoziation
	Dermatosklerose	Invers korreliert mit Vitamin-D-Spiegel
	ILD	Hypoxie, Immobilisation
	Calcinosis cutis	Unabhängiger Marker für Osteoporose
Medikamentös	Protonenpumpeninhibitoren	Beeinträchtigten Kalziumresorption
	Glukokortikoide	Auch bei niedrigeren Dosen als bei RA nachteilig für Knochengesundheit

*SSc* Systemische Sklerose, *DU* Digitale Ulzera, *ILD* Interstitielle Lungenerkrankung, *RA* Rheumatoide Arthritis

#### Vaskulopathie

Eine gestörte Mikrozirkulation, insbesondere in Form digitaler Ulzerationen (DU) und fortgeschrittener kapillarmikroskopischer Veränderungen, zeigt eine signifikante Assoziation mit reduzierter KMD sowie Störung der trabekulären Mikroarchitektur [3, 18–20]. Die mikroangiopathische Schädigung führt zu einer verminderten Perfusion des Knochengewebes, wodurch die osteoblastäre Aktivität gehemmt und die Knochenresorption gefördert wird. Durch die resultierende Dysregulation der Knochenhomöostase steigt das Risiko für Osteoporose an [18, 19].

#### Autoimmunität

Sowohl Anti-Zentromer-Antikörper (ACA) als auch Anti-Topoisomerase-I-Antikörper (Anti-Scl-70) wurden in Fall-Kontroll-Studien mit einer verminderten KMD in Verbindung gebracht. Marot et al. beschrieben eine signifikante Assoziation zwischen ACA und einer reduzierten KMD basierend auf Dual-Röntgen-Absorptiometrie (DXA)-Messungen [21]. Horváth et al. wiesen erstmals einen Zusammenhang zwischen dem Vorliegen von Anti-Scl-70-Antikörpern und einer verringerten KMD nach, gemessen mittels peripherer quantitativer Computertomographie (pQCT) am Radius der dominanten Hand [20]. Zudem zeigten sich bereits in einer älteren Studie erhöhte Werte des Knochenabbaumarkers Beta-Crosslaps (CTX) bei Anti-Scl-70-positiven Patienten [22].

Im Unterschied zur RA, bei der Autoantikörper wie ACPA direkt osteoklastäre Signalwege modulieren [23], liegen für SSc bislang überwiegend Assoziationsdaten vor. Direkte pathophysiologische Effekte von Autoantikörpern der SSc auf den Knochenstoffwechsel sind bisher nicht nachgewiesen.

#### Hypovitaminose D und Dermatosklerose

Eine Hypovitaminose D (25-OH-Vitamin D < 30 ng/ml) ist bei SSc sehr häufig, wobei Defizite in bis zu 97 % der unbehandelten Patienten nachgewiesen wurden [24]. Die Vitamin-D-Spiegel sind besonders niedrig bei Patienten mit diffuser Hautbeteiligung und korrelieren invers mit dem Ausmaß der Dermatosklerose, GI-Beteiligung und dem Schweregrad der Erkrankung [24–26]. Die inverse Assoziation zwischen Dermatosklerose und 25 (OH) D dürfte primär durch eine verminderte UV-B-Penetration fibrotischer Haut sowie mikrovaskuläre Einschränkungen der kutanen Synthese erklärt werden [25]. Ein niedriger 25-OH-D-Serumspiegel korreliert signifikant mit verringerter KMD sowie erhöhten Knochenstoffwechselparametern. Ein Vitamin-D-Mangel ist mit einer höheren Osteoporoseprävalenz, reduzierter trabekulärer und kortikaler KMD und einer gesteigerten Frakturrate assoziiert [20, 25, 26].

#### Calcinosis cutis

Mehrere multizentrische Studien belegen eine signifikante Assoziation zwischen Calcinosis cutis und Osteoporose bei Patienten mit SSc (OR bis 4,2). Diese Beziehung ist nicht kausal, sondern spiegelt die gemeinsame Pathophysiologie schwerer Krankheitsverläufe wider: Calcinosis cutis tritt gehäuft bei Patienten mit ausgeprägter vaskulärer Dysfunktion und entzündlicher Aktivität auf – Zustände, die ebenfalls mit einer erhöhten Osteoporoseprävalenz einhergehen. Als Marker für eine schwere Krankheitsausprägung kann Calcinosis cutis somit indirekt auf ein erhöhtes Frakturrisiko hinweisen. Die Assoziationen bestehen unabhängig von klassischen Risikofaktoren wie Alter, Menopausenstatus oder BMI [27, 28].

#### Interstitielle Lungenerkrankung (ILD)

Eine ILD bei der SSc ist häufig. Diese war als unabhängiger Risikofaktor in einer Fall-Kontroll-Studie mit einer reduzierten KMD assoziiert [20]. Die bei ILD häufig bestehende chronische Hypoxämie und die eingeschränkte körperliche Belastbarkeit könnten pathogenetische Faktoren darstellen, die zur Beeinträchtigung der KMD beitragen [29]. Andernfalls könnte die ILD ähnlich wie die Calcinosis cutis Marker eines schwereren Verlaufs sein, wobei die Assoziation zur KMD auch unabhängig von weiteren Faktoren wie DU bestand.

## Diagnostik

Der Goldstandard der Diagnostik stellt wie bei anderen Formen der Osteoporose die Messmethode mittels DXA an der Lendenwirbelsäule und am gesamten Schenkelhals dar. Wie bereits zuvor erwähnt, weisen Patienten mit SSc bereits frühzeitiger eine Knochendichteminderung auf als die Allgemeinbevölkerung. Zudem sind Männer ebenfalls betroffen, was auf ein geschlechtsunabhängiges osteologisches Risikoprofil hinweist [3]. Darüber hinaus finden sich in dieser Patientengruppe signifikante Veränderungen der Knochenstoffwechselparameter wie Osteocalcin und Desoxypyridinoline im Serum und Urin [26]. Die Indikation zur Knochendichtemessung (mittels DXA) bei Patienten mit SSc ist aufgrund der Häufung klassischer Risikofaktoren wie auch zusätzlichen krankheitsassoziierter Risikofaktoren somit insgesamt großzügiger zu stellen [30].

Wie in Studien gezeigt, weist die Knochenmikroarchitektur bei der SSc eine besondere Vulnerabilität auf. Ein geeignetes Messinstrument diesbezüglich stellt der Trabecular Bone Score (TBS) dar, welcher bei Patienten mit SSc signifikant niedriger als in der Allgemeinbevölkerung und vergleichbar zu Patienten mit RA ausfällt [17]. Der TBS kann dazu beitragen, das Frakturrisiko besser einzuschätzen, da die alleinige DXA-Messung die Beeinträchtigung der Knochenstruktur unterschätzen kann. Zudem ist der TBS ein von der KMD unabhängiger Prädiktor für Frakturen [31]. Die Integration von TBS in die Frakturrisikobewertung wird von internationalen Expertengruppen wie der European Society for Clinical and Economic Aspects of Osteoporosis, Osteoarthritis and Musculoskeletal Diseases (ESCEO) und der International Osteoporosis Foundation (IOF) ausdrücklich empfohlen, um die Risikostratifizierung bei sekundärer Osteoporose (wie der SSc) zu verbessern [32].

Eine TBS-Messung sollte bei SSc-Patienten insbesondere dann durchgeführt werden, wenn die DXA-Messung allein keine eindeutige Aussage zum Frakturrisiko erlaubt. Ein niedriger TBS (< 1,20) weist auf eine relevante Mikroarchitekturstörung mit erhöhtem Frakturrisiko hin, während höhere Werte (> 1,35) eine strukturelle Beeinträchtigung weniger wahrscheinlich machen [32]. In Deutschland wird TBS im Rahmen der DVO-Leitlinie ebenfalls in die Risikobewertung einbezogen, wobei – im Gegensatz zur DXA-basierten T‑Wert-Betrachtung – die Einordnung in Abhängigkeit vom altersadjustierten Z‑Wert erfolgt [6]. In einer aktuellen Studie bei Patienten mit SSc wies der TBS eine höhere Genauigkeit zur Detektion vertebraler Frakturen auf, als die alleinige DXA-basierte Messung der KMD [33]. Vor diesem Hintergrund erscheint die Ergänzung der Diagnostik um eine TBS-Messung sinnvoll, insbesondere bei unklarer Risikokonstellation – wenngleich die Verfügbarkeit entsprechender Messsysteme in Deutschland derzeit noch eingeschränkt ist.

## Therapie

Voraussetzung für die Homöostase des Knochenstoffwechsels ist eine ausreichende Versorgung mit Kalzium und Vitamin D. Bei einem Großteil der Patienten mit SSc liegt eine Hypovitaminose D vor [24]. Diese stellt einen modifizierbaren Risikofaktor für die Entstehung von Osteoporose dar und sollte entsprechend behoben werden. Die allgemeine Empfehlung der Substitution von 800 IE pro Tag reicht in den meisten Fällen nicht aus. Die Höhe der Vitamin-D-Supplementation sollte sich nach den laborchemisch gemessenen Spiegeln orientieren und mindestens 30 ng/ml betragen (maximal 100 ng/ml) [30]. Bezüglich der Kalziumzufuhr liegen für Patienten mit SSc derzeit keine spezifischen Empfehlungen vor. Aufgrund der häufigen Langzeittherapie mit PPI und der Malnutrition ist das Risiko einer Hypokalzämie bei SSc-Patienten erhöht, weswegen eine Supplementation zu erwägen ist [13, 16]. Die Supplementierung sollte sich an den allgemeinen Leitlinien zur Osteoporose orientieren, wobei eine tägliche Aufnahme von etwa 1000 mg Kalzium angestrebt wird [6]. Gastrointestinale Nebenwirkungen wie Obstipation, Blähungen oder abdominelle Beschwerden können insbesondere bei höheren Kalziumdosen oder bei vorbestehender gastrointestinaler Motilitätsstörung – wie sie bei SSc häufig ist – auftreten [14]. Die Verträglichkeit sollte deshalb individuell geprüft und die Dosis gegebenenfalls angepasst werden. Nach aktueller Datenlage besteht kein Hinweis darauf, dass eine Kalziumsupplementierung das Risiko für die Entwicklung einer Calcinosis cutis bei SSc erhöht [30].

Eine besondere Herausforderung der osteospezifischen Therapie bei SSc stellt insbesondere die hochprävalente gastrointestinale Beteiligung dar, die sowohl die Resorption als auch die Verträglichkeit oraler Medikamente einschränken kann [14].

Orale Bisphosphonate sind eine etablierte Therapie der Osteoporose und senken nachweislich die Frakturrate. Ihre orale Bioverfügbarkeit ist jedoch auf 1–6 % begrenzt und kann bei Malabsorption oder verzögerter Magenentleerung, wie z. B. bei SSc-bedingter Gastroparese, weiter reduziert sein. Zudem erhöht ein verlängerter Kontakt mit der oberen gastrointestinalen Schleimhaut, wie z. B. bei der SSc-bedingten Refluxerkrankung bzw. der Magenentleerungsstörung, das Risiko mukosaler Irritationen und Ulzerationen [34, 35]. Des Weiteren weisen Patienten mit SSc eine höhere Prävalenz parodontaler Erkrankungen auf (81,6–90,7 % vs. 48,8–71,1 % in Kontrollen; OR 7,63, 95 % KI [1,7; 33,5]) [36]. Krankheitsassoziierte Faktoren wie Xerostomie infolge fibrotischer Speicheldrüsenveränderungen, Mikrostomie mit eingeschränkter Mundhygiene und eine vaskuläre Dysfunktion im Bereich der oralen Mikrozirkulation tragen wesentlich zu dieser erhöhten Anfälligkeit bei [14, 36]. Diese Faktoren begünstigen bakterielle Plaquebildung, beeinträchtigen die orale Immunabwehr und führen zu einer gestörten Geweberegeneration. Da die Entstehung einer aseptischen Kiefernekrose maßgeblich durch gestörte Vaskularisation, lokale Hypoxie und eingeschränkte Heilung nach zahnärztlichen Eingriffen bestimmt wird [37], können die bei SSc vorliegenden Veränderungen dieses Risiko zusätzlich verstärken. Parodontalerkrankungen wirken zwar nicht direkt ursächlich, können jedoch über die beeinträchtigte Wundheilung zur Entwicklung einer Kiefernekrose unter antiresorptiver Therapie beitragen [37]. Daher ist bei SSc-Patienten ein sorgfältiges zahnärztliches Management vor und während antiresorptiver Behandlung besonders wichtig.

Erst kürzlich wurde eine Pilotstudie zur Effektivität und Sicherheit antiresorptiver Substanzen bei Patienten mit SSc und Osteoporose veröffentlicht [38]. Hier zeigte sich eine vergleichbare Wirksamkeit von Bisphosphonaten und Denosumab für Patienten mit SSc im Vergleich zu publizierten Daten bei postmenopausaler Osteoporose. Bezüglich der Sicherheit entsprachen die beobachteten insbesondere gastrointestinalen Nebenwirkungen weitgehend dem bekannten Profil der postmenopausalen Osteoporose, traten alle bei Patienten mit nachgewiesener ösophagealer Hypotonie auf und wurden allesamt als nicht schwerwiegend eingestuft. Allerdings trat eine aseptische Kiefernekrose unter Therapie mit Ibandronat auf, was die Notwendigkeit einer sorgfältigen Nutzen-Risiko-Abwägung bei der Wahl der antiresorptiven Therapie – insbesondere bei bestehender oraler Komorbidität unterstreicht.

Aufgrund der geringen Patientenanzahl (*n* = 42) und des retrospektiven Designs sind die Daten mit Vorsicht zu interpretieren.

Daten zur osteoanabolen Therapie mit Teriparatid, Romosozumab oder Abaloparatid liegen nicht vor. Bei manifester Osteoporose oder sehr hohem Frakturrisiko sollten diese Substanzen aus Sicht der Autoren auch bei der SSc eingesetzt werden, sofern keine Kontraindikationen vorliegen.

Zu berücksichtigen ist, dass anders als bei der Frau beim Mann zur Behandlung der Osteoporose ausschließlich Alendronat (10 mg täglich), Risedronat (35 mg wöchentlich), Zoledronat (5 mg jährlich i.v.), Denosumab (60 mg halbjährlich s.c.) sowie Teriparatid (20 µg täglich s.c.) zugelassen sind.

Anders als beispielsweise bei der RA, wo in prospektiven Studien ein osteoprotektiver Effekt insbesondere der zytokinbasierten krankheitsmodifizierenden Basistherapie (primär über eine effektive Krankheitskontrolle) nachgewiesen werden konnte, fehlt diese Evidenz bei der SSc [39]. Zu krankheitsmodifizierenden Therapien bei SSc (Rituximab, Tocilizumab, Mycophenolat-Mofetil), Antifibrotika (z. B. Nintedanib) und autologer Stammzelltransplantation liegen bislang keine prospektiven Daten mit knochenspezifischen Endpunkten (BMD, TBS, Frakturen) vor.

Insgesamt ist die Evidenz zur osteospezifischen Therapie bei SSc bislang fragmentarisch, überwiegend aus retrospektiven Analysen und Querschnittsstudien abgeleitet und durch kleine Patientenzahlen limitiert. Vor diesem Hintergrund erscheint die Entwicklung krankheitsspezifischer Leitlinien zur Prävention, Diagnostik und Therapie der Osteoporose bei entzündlich-rheumatologischen Erkrankungen, wie auch der SSc, wünschenswert.

## Fazit für die Praxis

Patienten mit SSc weisen ein erhöhtes Risiko für Osteoporose und Frakturen auf. Neben einer Häufung klassischer Risikofaktoren tragen insbesondere Vaskulopathie, Fibrose und gesteigerte Autoimmunität wesentlich zur Knochenschädigung bei. Eine frühzeitige DXA-Messung und ggf. TBS-Ergänzung sollte auch bei prämenopausalen Frauen und Männern großzügig erfolgen. Aufgrund potenziell eingeschränkter Resorption und Verträglichkeit oraler Bisphosphonate sind parenterale Antiresorptiva wie Denosumab eine potenziell geeignetere Alternative. Ein individualisiertes, risikoadaptiertes Vorgehen ist essenziell.
